# Result on speech perception after conversion from Spectra® to Freedom®

**DOI:** 10.1590/S1808-86942012000200003

**Published:** 2015-10-20

**Authors:** Ana Tereza de Matos Magalhães, Maria Valéria Schmidt Goffi-Gomez, Ana Cristina Hoshino, Robinson Koji Tsuji, Ricardo Ferreira Bento, Rubens Brito

**Affiliations:** a MSc student – FMUSP (Speech and Hearing Therapist of the Cochlear Implant Team of the University Hospital – Medical School of the University of São Paulo – HC-FMUSP); b PhD in Sciences – Federal University of São Paulo (Speech Therapist of the Otorhinolaryngology Clinic at the University Hospital of the Medical School of the University of São Paulo – USP; Coordinator of the Speech and Hearing Therapy team of the Cochlear Implant Team of the HC-FMUSP); c PhD Student – Federal University of Rio de Janeiro (Speech and Hearing Therapist); d PhD – FMUSP (ENT physician – University Hospital – USP; Coordinator of the Cochlear Implant Team – HC-FM-USP); e Full Professor of the Ophthalmology and Otorhinolaryngology Department of the Medical School of the University of São Paulo (FMUSP) – Otorhinolaryngology Course (Head of the Ophthalmology and Otorhinolaryngology Department – FMUSP); f Senior Associate Professor of Otorhinolaryngology Program of the Medical School of the University of São Paulo (FMUSP); Assistant Physician of the Otorhinolaryngology Department of the University Hospital of the University of São Paulo)

**Keywords:** adult, audiometry, cochlear implants, speech discrimination tests, speech perception

## Abstract

New technology in the Freedom® speech processor for cochlear implants was developed to improve how incoming acoustic sound is processed; this applies not only for new users, but also for previous generations of cochlear implants.

**Aim:**

To identify the contribution of this technology – the Nucleus 22® – on speech perception tests in silence and in noise, and on audiometric thresholds.

**Methods:**

A cross-sectional cohort study was undertaken. Seventeen patients were selected. The last map based on the Spectra® was revised and optimized before starting the tests. Troubleshooting was used to identify malfunction. To identify the contribution of the Freedom® technology for the Nucleus22®, auditory thresholds and speech perception tests were performed in free field in soundproof booths. Recorded monosyllables and sentences in silence and in noise (SNR = 0dB) were presented at 60 dBSPL. The nonparametric Wilcoxon test for paired data was used to compare groups.

**Results:**

Freedom® applied for the Nucleus22® showed a statistically significant difference in all speech perception tests and audiometric thresholds.

**Conclusion:**

The reedom® technology improved the performance of speech perception and audiometric thresholds of patients with Nucleus 22®.

## INTRODUCTION

The first Brazilian multichannel cochlear implant surgeries were done in 1999^1^, using the *Spectra Bodyworn* speech processor, from Cochlear Corporation®, which works well with the *Nucleus 22*® internal unit.

Throughout the years, new Technologies have been launched and cochlear implant companies developed speech processors for the previous internal units, avoiding the need for a new surgical procedure^2^, ^3^, ^4^.

In 2006, the *Freedom*® processor was launched, with new Technologies, among which two microphones and a new chip for signal processing, providing improvements in the processing of the input acoustic sound, not only for new users but also for patients with previous generations of cochlear implant.

In 2009, the replacement parts for the *Nucleus 22*® system were no longer manufactured. The Ministry of Health, then, authorized the exchange, through the Brazilian Public Health Care System (SUS), of all the *Nucleus 22*® speech processors for the *Freedom*® speech processors.

In the scientific literature, numerous studies have shown the advantage in exchanging it for more modern processors; nonetheless, the direct exchange of the *Spectra*® for the *Freedom*® was not studied, nor reported^3^, ^5^.

Therefore, this study aimed at identifying the contribution of the *Freedom*® speech processor technology for the first generation of the *Nucleus 22*® *multichannel cochlear implant.* For that, we assessed the speech perception performance in silence and in noise and the audiometric thresholds with the *Spectra*® and *Freedom*® *speech processors*.

## METHODS

This Project was presented to and approved by the Ethics Committee for the Analysis of Research projects of the institution under protocol number 0083/11. The patients were informed about the research and signed the informed consent form.

The study was a cross-sectional historical cohort, assessing the results obtained with the update of the speech processor technology of the *Nucleus 22*® *cochlear implant*.

We selected 43 patients implanted with the *Nucleus 22*® in the Cochlear Implant Group of the University Hospital of the Medical School of the University of São Paulo (USP). This way, five patients were no longer users of the cochlear implant, eight patients were already users of the *Freedom*® processor. The remaining 30 patients kept using the *Spectra*® processor and came to change the speech processor, offered by the Brazilian Government through the Ministry of Health.

In the study, we included the patients who were effectively using the implant (8 hours per day), and we took off the study those patients using it occasionally or those who did not have speech perception in the closed context. Six patients were not effective users, four patients were children and three patients did not have speech perception. Ultimately, the study included 17 patients.

The patients called followed the following protocol, broken down into three stages:

### a First stage: optimization of the current map and checking how the *Spectra*® processor worked

At this first stage, the last map being used by the patient with the *Spectra*® processor was reviewed and updated according to the programming routine with the *Custom Sound 2.0*® software. We studied the minimum stimulation levels (level T), in which the patient identifies 100% of the presentations; the maximum levels of stimulation (level C) for those whom the electric current presented is comfortable and the level C intensity was balanced. Before doing the tests, the *Spectra*® processor functioning was checked, inspecting the wires, antenna transmission and microphone, using a signal checking device and the monitoring phone. Should any fault be found, the component would be replaced before the tests.

### b Second stage: programming the *Freedom* processor

We exchanged the *Freedom*® processor, maintaining the other parameters of the original map: total stimulation velocity and stimulation mode, without a signal pre-processing strategy. We balanced the intensity of the T and C stimulation levels with the *Custom Sound 2.0*® software.

### c Third stage: assessing the audiometric thresholds and the speech perception tests

We carried out free field audiometry, with the *Spectra*® and the *Freedom*® speech processors. In order to do the statistical calculation, we considered the mean values of the audiometric thresholds at 500 Hz; 1,000 Hz; 2,000 Hz and 4,000 Hz^6^ and the frequency thresholds individually.

In the same session, after programming all the maps, the following free field speech perception tests were carried out in a sound-treated booth, with the material recorded in a CD, presented at 60dBSPL^7^:

• Monossylables^8^, ^9^;
• Open phrases in the silence^10^;
• Open phrases in noise with SNR = 0dB* ^10^

The order in which the tests with the *Spectra*® processor maps and the new maps with the *Freedom*® processor was randomized using the *software* available at www.randomizer.org

The audiometric thresholds, percentage of correct answers in the speech perception tests are presented as means and standard deviation (SD) and also as median and the percentiles 25% and 75% (P25% and P75%, respectively). Considering the small sample size, the ordinal nature and asymmetrical distributions of some variables, the Wilcoxon non-parametric test for paired data was utilized in order to compare the groups. The analysis of the parametric sensitivity utilizing the t test was carried out in all the comparisons and it did not change the results. A *p* < *0.05* was considered as statistically significant.

The statistical analyses were carried out using the STATA software (version 11.1).

## RESULTS

Table 1 depicts the demographic data of the sample.

**Table 1:** Sample demographic data.

Patients	Gender^a^	Age (years)	Etiology	Deafness duration (years)	Duration of CI^b^ Use	Number of active electrodes in the map (years) (active channels)
S1	M	50	Progressive	15	8	15 (17)
S2	M	68	Progressive	10	9	16 (18)
S3	F	47	Chicken pox	23	9	16(18)
S4	F	63	Otosclerosis	5	10	14 (16)
S5	M	82	Unknown	2	11	20 (20)
S6	M	49	Head Injury	2	10	20 (20)
S7	M	69	Progressive	3	9	18 (20)
S8	F	43	Meningitis	1	11	16 (18)
S9	F	15	Congenital	7	8	18 (20)
S10	M	29	Progressive	4	10	20 (20)
S11	F	47	Meningitis	28	10	20 (20)
S12	F	43	Meningitis	27	8	18 (20)
S13	M	58	Head Injury	2	8	18 (20)
S14	M	17	Meningitis	1	8	5 (5)
S15	F	30	Unknown	4	9	18 (20)
S16	M	50	Unknown	11	11	20 (20)
S17	F	44	Unknown	9	8	19 (19)

a M – male; F – female;

b CI – cochlear implant.

Most of the sample is made up of patients with more than eight years of experience using the cochlear implant. Only two were teenagers, and one of them had congenital deafness.

When we analyzed the contribution of the *Freedom*® for patients using the *Nucleus 22*®, we noticed a statistically significant difference in all the speech perception tests and in all audiometric thresholds, both individually as in the mean, except in 8,000 Hz (Tables 2 and 3).

**Table 2:** Speech perception results with both speech processors.

	Spectra®			Freedom®			
	Median (P25%, P75%^c^)	Mean (SD^d^)	n^e^	Median (P25%, P75%c)	Mean (SD^d^)	ne	*p*^f^(Wilcoxon)
Monosyllables	16 (4, 32)	20.9 (18.2)	17	36 (20, 56)	34.8 (19.9)	17	0.0011*
Phrases in noise	0 (0, 60)	26.5 (32.8)	17	40 (10, 80)	46.5 (36.7)	17	0.0021*
Phrases in silence	20 (0, 30)	20.0 (18.7)	9	60 (60, 70)	56.7 (26.0)	9	0.0174*

c P25% – 25% percentage; P75% – 75% percentage;

d SD – standard deviation;

e n – number of patients;

f *p* < 0.05

(*) – statistically significant result.

**Table 3:** Results from the audiometric thresholds with both speech processors.

	Spectra®			Freedom®			
	Median (P25%, P75%^c^)	Mean (DP^d^)	n^e^	Median (P25%, P75%^c^)	Mean (DP^d^)	n^e^	*p*^f^(Wilcoxon)
250 Hz	50 (45, 55)	49.4 (13.3)	17	35 (30, 40)	35.0 (9.8)	17	0.0004*
500 Hz	55 (45, 55)	52.1 (7.5)	17	40 (35, 40)	37.6 (5.6)	17	0.0004*
1000 Hz	45 (45, 50)	47.4 (6.6)	17	30 (25, 35)	29.7 (6.2)	17	0.0002*
1500 Hz	45 (40, 50)	46.5 (8.4)	17	30 (25, 35)	30.0 (8.8)	17	0.0003*
2000 Hz	50 (40, 55)	47.6 (8.9)	17	30 (25, 35)	31.2 (8.2)	17	0.0003*
3000 Hz	50 (45, 55)	49.1 (8.9)	17	35 (25, 40)	33.5 (9.3)	17	0.0005*
4000 Hz	55 (45, 55)	53.5 (9.5)	17	45 (30, 45)	38.5 (9.1)	17	0.0004*
6000 Hz	55 (45, 65)	58.2 (12.9)	17	40 (30, 50)	41.2 (16.3)	17	0.0007*
8000 Hz	60 (50, 75)	63.5 (15.2)	17	80 (70, 90)	74.7 (16.4)	17	0.0084*
BIAP^g^	48.7 (45, 53.7)	50.1 (6.3)	17	35 (28.7, 38.7)	34.3 (5.9)	17	0.0003*

c P25% – 25% percentage; P75% – 75% percentage;

d SD – standard deviation;

e n – number of patients;

f p < 0.05

(*) – statistically significant result;

g BIAP, 1996 – mean value of the 500 Hz, 1000 Hz, 2000 Hz and 4000 Hz thresholds.

## DISCUSSION

Exchanging the *Spectra*® processor for the *Freedom*® proved that the technology improved the speech perception of *Nucleus 22*® users. Even when maintaining the same parameters of the stimulation mode, the total velocity and number of active electrodes, we can see a statistical difference in all the tests and in all the audiometric thresholds (Tables 2 and 3).

The scientific literature has shown that there are advantages when the speech processor is exchanged for more modern devices. Dodd et al.^3^ published their results concerning the exchange of the *Spectra*® box processor for the *ESPrit*® behind-the-year processor in 100 children; and they concluded that the conversion was feasible in all the cases studied, although the speech tests and audiometric thresholds did not show significant differences.

Santarelli et al.^5^ assessed the speech perception in 17 prelingual children users of the *Nucleus 24*® with the *SPrint*® and *ESPrit 3G*® processors, after one month using the *Freedom*® processor. As results, the children had a significant improvement in the dissyllable words in the presence of noise, identification of consonants and phrase recognition. These improvements in phonemic discrimination may be explained by an increase in the input dynamic field with the new processor. They concluded that the speech perception was higher with the *Freedom*®, being advisable to exchange for the new generation of processors. Moreover, all the children preferred the new processor.

This shows the importance and the care taken by cochlear implant manufacturers in developing new processors, matching the internal units of previous generations, so that these patients may enjoy the new technology and, consequently, improve their performance, but also enable more flexibility in programming, keeping the characteristics and the preference of the old program used by the patient.

The possibility of keeping the same parameters of the old processor in use shows that only changes to the microphone and the signal processing have been enough to the processor's contribution in improving speech perception.

One of the parameters maintained was the stimulation mode. When one electrode is stimulated, the current flows from the active electrode to the ground one; and the broader the spacing between the electrodes the lower the necessary current.

Although the *Freedom*® has the BP+3 (bipolar, one active electrode and another as indifferent) stimulation mode as a standard for users of the *Nucleus 22*®, all the patients could maintain their modes of stimulation. With the aim of analyzing only the benefit of all the parameters studied, without harming the use of batteries, the stimulation modes utilized were maintained, i.e. BP+1 for all, except one patient who used the CG mode (*common ground* uses all the electrodes as different electrodes). Battery use was measured by the *Custom Sound 2.0*® *software*, finding an average of 8.6 hours for rechargeable batteries and 24.1 hours for the three 675 batteries. There were only two patients who had problems with battery consumption, with only 6 hours of battery life, which was solved by changing the power of the antenna magnet.

In this same sample, other technological resources were analyzed, as the effect of the frequency allocation table and the T-SPL effect of 25dB and that of the C-SPL of 65dB, which will be published later.

## CONCLUSION

The *Freedom*® processor technology for users of the *Nucleus 22*® showed a statistically significant improvement in all speech tests in silent environments at 60 dB and in noise, with a signal/noise ratio of 0 dB, as well as in all audiometric thresholds.

## Footnotes

* These tests were only employed when the phrase recognition performance in silence was higher than 50%.
